# An intensive education program for caregivers ameliorates anxiety, depression, and quality of life in patients with drug-resistant temporal lobe epilepsy and mesial temporal sclerosis who underwent cortico-amygdalohippocampectomy

**DOI:** 10.1590/1414-431X20209000

**Published:** 2020-07-17

**Authors:** Yuena Wang, Dongyu Hou, Xiaohua Wu, Lili Qiu, Hong Chen, Jianxia Xin, Zhirong Yan, Meiling Sun

**Affiliations:** 1 Department of Neurosurgery, The 2nd Affiliated Hospital of Harbin Medical University, Harbin, China; 2 Department of Orthopedics, The 2nd Affiliated Hospital of Harbin Medical University, Harbin, China; 3 Department of Endocrinology, The 2nd Affiliated Hospital of Harbin Medical University, Harbin, China; 4 Department of Nursing, The 2nd Affiliated Hospital of Harbin Medical University, Harbin, China

**Keywords:** Refractory temporal lobe epilepsy and mesial temporal sclerosis, Cortico-amygdalohippocampectomy, Caregiver intensive education program, Anxiety, Depression, Quality of life

## Abstract

This study aimed to investigate the effect of a caregiver intensive education program (CIEP) on anxiety, depression, and quality of life (QOL) in patients with drug-resistant temporal lobe epilepsy and mesial temporal sclerosis (TLE-MTS) who underwent cortico-amygdalohippocampectomy (CAH). Ninety patients with drug-resistant TLE-MTS who underwent CAH and their caregivers were recruited and randomly allocated to the CIEP group or control group as 1:1 ratio. Caregivers received the CIEP program or routine guidance/education (control group). Anxiety/depression and QOL in patients at month (M)0, M1, M3, and M6 were assessed by the Hospital Anxiety and Depression Scale (HADS) scale and the QOL in Epilepsy Inventory-31 (QOLIE-31), respectively. Treatment efficacy at M6 was assessed by Engel classification. The HADS-anxiety score at M3 (P=0.049) and M6 (P=0.028), HADS-anxiety score change (M6-M0) (P=0.001), percentage of anxiety patients at M6 (P=0.025), and anxiety severity at M6 (P=0.011) were all decreased in the CIEP group compared with the control group. The HADS-depression score at M6 (P=0.033) and HADS-depression score change (M6-M0) (P=0.022) were reduced, while percentage of depression patients at M6 (P=0.099) and depression severity at M6 (P=0.553) showed no difference in the CIEP group compared with the control group. The QOLIE-31 score at M6 (P=0.043) and QOLIE-31 score change (M6-M0) (P=0.010) were both elevated in the CIEP group compared with the control group. In conclusion, CIEP for caregivers contributed to the recovery of anxiety and depression as well as the improvement of QOL in patients with drug-resistant TLE-MTS who underwent CAH.

## Introduction

Epilepsy is a frequent brain disease caused by a disorder of the excitatory and inhibitory balance in the neuronal network, affecting more than 70 million people worldwide (1). Temporal lobe epilepsy and mesial temporal sclerosis (TLE-MTS) is one of the most common types of epilepsy in clinical practice (2). The refractory disease caused by drug resistance is a crucial issue in TLE-MTS treatment, and although there is strong evidence showing that surgery is effective in treating refractory TLE-MTS, a high prevalence of post-surgery psychiatric disorder still occurs, which hampers the management of patients and blocks the way to a full recovery (3–5). Therefore, management of psychiatric disorders in patients with drug-resistant TLE-MTS who underwent surgery is urgently required.

In addition, the caregivers, mostly consisting of families, relatives, and friends, also play a determinant role in improving epilepsy patient outcomes. There is a report disclosing that factors related to caregivers, such as poor family relationship, caregiver depression, and caregiver unemployment could predict the patients' mental health (6). Therefore, an intervention with caregivers aiming to avoid the caregiver-related pejorative factors that harm the recovery of psychiatric disorders in TLE-MTS patients is necessary. There have been several reports demonstrating that special care programs might benefit epilepsy patients regarding ameliorating anxiety and depression as well as improve the quality of life (QOL); however, very few studies have been done to explore the effect of care programs for caregivers in ameliorating the psychiatric disorders in epilepsy patients (7,8). Moreover, to the best of our knowledge, no study has been done to assess the care programs designed for caregivers in enhancing anxiety and depression recovery as well as improving QOL in patients with drug-resistant TLE-MTS post cortico-amygdalohippocampectomy (CAH) treatment.

Thus, the aim of this study was to investigate the effect of a caregiver intensive education program (CIEP), a professional, intensive, and comprehensive education program for caregivers, on anxiety, depression, and QOL of patients with drug-resistant TLE-MTS who underwent CAH.

## Material and Methods

### Participants

In this randomized controlled study, 90 patients with drug-resistant TLE-MTS who underwent CAH and their caregivers were consecutively recruited from the 2nd Affiliated Hospital of Harbin Medical University between January 2014 and December 2017. The inclusion criteria for patients were: i) confirmed diagnosis of refractory TLE based on International League Against Epilepsy (ILAE) classification (9) concomitant with unilateral MTS confirmed by magnetic resonance imaging examinations based on a protocol routinely used for patients with TLE, which included the following (axial and coronal slices): T2-weighted images, fluid-attenuated inversion-recovery (FLAIR) and proton density, T1 inversion recovery and sagittal volumetric sequences with contiguous slices, with and without administration of gadolinium (MR acquisition parameters were set as described in the previous study (10)); iii) underwent CAH; iv) aged more than 18 years; and v) had a permanent caregiver in their family (caregiver was defined as the family member who was primarily responsible for patient care). The exclusion criteria for patients with drug-resistant TLE-MTS included: i) bilateral MTS; ii) other neurological illnesses besides epilepsy; iii) complicated with other malignant diseases hindering clinical evaluations; iv) unable to comprehend the study contents or fulfill the questionnaires independently; v) unable to be followed up regularly, which was assessed by the investigator based on the patient's overall conditions; and vi) pregnant or breastfeeding women. In addition, caregivers of the patients were required to be in good health and have normal cognitive function, which was defined as Mini-mental State Examination (MMSE) score ≥26.

### Ethics statement

The current study was approved by the Ethics Committee of the 2nd Affiliated Hospital of Harbin Medical University and performed in accordance with the recommendations from the Declaration of Helsinki. All patients and their caregivers signed informed consents before study initiation.

### Randomization

Eligible patients were randomly allocated to the CIEP group or control group as a 1:1 ratio with 45 patients in each group using the blocked randomization method. The assignment of patients was performed according to the allocation sequence created by SAS 9.0 (SAS Institute, Inc., USA) by an independent nurse without involvement in the other parts of the study.

### Baseline data collection

Baseline data of patients were documented on the Case Report Form after enrollment, which included age at epilepsy onset, age at surgery, gender, marital status, highest education, employment status, hypertension, hyperlipidemia, diabetes, family history of epilepsy, family history of psychiatric disorders, disease duration, seizure frequency, presence of febrile seizures, presence of left-sided MTS, slow video‐electroencephalographic (VEEG) background activity, asymmetric VEEG background activity, and contralateral slow waves on VEEG. In addition, caregivers' demographic information including age, gender, and highest education was recorded.

### Intervention

After surgery, all patients and their caregivers received conventional medical treatment, guidance, as well as care (such as care for surgical wound, prevention of head injury, long-term drug management, epilepsy seizure management, psychological care, diet management, exercise, rest, etc). All patients were invited to the hospital for re-examination and corresponding assessments at 1 month after CAH (M1), M3, and M6, during which rehabilitation guidance was given to them according to the status of patients.

In the CIEP group (n=45), a brief introduction of CIEP was given to the patients and their caregivers at the first week after enrollment, and the education materials and a booklet for documenting medicines and seizures were distributed. Then CIEP was carried out two times per month by inviting the caregivers to the Rehabilitation Center. CIEP consisted of 12 educational sessions, which were delivered every two weeks, 90 min each time, and lasted for 6 months. The educational sessions were delivered in group sessions (2–3 patients in a group) by the trained nurses in face-to-face lectures, and each educational session included four parts: part 1 (about 40 min), trained nurses gave the detailed instructions and explanations to the caregivers according to the 12 themes in the education materials (as shown in the Table 1); part 2 (about 30 min), the trained nurses communicated with caregivers to know about the problems or barriers they met in daily patient care, then discussed the solution strategies together; part 3 (about 15 min), trained nurses checked the records of medicine use and epilepsy seizure status documented in the booklet and gave appropriate guidance about patient management of medicines and seizures to the caregivers; part 4 (about 5 min), the nurse was responsible for scheduling appointments with the doctor according to the individual needs.

In the control group (n=45), detailed nursing guidance and education was given to patients and caregivers by nurses at discharge from the hospital. The education materials and the booklet for documenting medicines and seizures were also given to them. Other appropriate guidance was given to the patients and caregivers when they returned to the hospital for assessment (at M1, M3, and M6).

### Assessment of anxiety, depression, and QOL

The anxiety and depression of patients were assessed using the Hospital Anxiety and Depression Scale (HADS) at baseline (M0), M1, M3, and M6, and the QOL was also evaluated at these time-points with the use of the QOL in Epilepsy Inventory-31 (QOLIE-31, Version 1.0). All scales were independently filled out by the patients at each time-point, and there was an independent nurse responsible for the collection of the scales and the calculation of HADS-anxiety (HADS-A) score, HADS-depression (HADS-D) score, and QOLIE-31 score. The HADS consists of seven questions scored as 0–3 points individually, resulting in a total of 0–21 points and being classified as: 0–7, no anxiety/depression; 8–10, light anxiety/depression; 11–14, moderate anxiety/depression; 15–21, severe anxiety/depression (11). The QOLIE-31 contains seven multi-item scales that cover the following health concepts: well-being, social functioning, energy/fatigue, cognitive functioning, seizure worry, medication effects, and overall QOL. A QOLIE-31 overall score was obtained using a weighted average of the multi-item scale scores, and higher scores reflected better QOL and lower scores indicated worse QOL (12).

### Assessment of treatment efficacy

Therapy efficacy was assessed by the treating physicians using Engel classification (13), which was performed at M6 by a thorough review of the patient's seizure diaries and medical files and by considering the changes in seizure frequency, seizure intensity, and the impact of these changes on the patient’s QOL. Class I was defined as a patient free of disabling seizures; class II, patients with rare seizures at a frequency of three or less per year; class III outcome corresponded to a “worthwhile” result occurring through a reduction either in seizure frequency or seizure intensity that improved the patient's QOL; class IV was defined as seizure frequency was not reduced, or reduced only to such limited extent that it did not improve day-to-day functioning.

### Sample size calculation

To detect a significant difference in anxiety rate at M6 between the two groups, we assumed that the anxiety rate at M6 was 20% in the CIEP group and 50% in the control group. Using a power of 80% and a two-sided 5% level of significance (α), a sample size of 39 patients in each group was required. Accounting for dropouts and loss to follow-up of about 15%, the sample size was increased to 90 with 45 patients in each group.

### Statistical analysis

All 90 patients were included in the final analysis based on the intention-to-treat (ITT) principle with the last observation carried forward (LOCF) method from any of the three post-baseline measures. Data analyses were performed with SPSS 20.0 software (IBM, USA) and GraphPad Prism 6.01 (GraphPad Software Inc., USA). Continuous data are reported as means±SD, and the comparison between two groups were done by Student's *t*-test. Count data are reported as count (percentage), and the comparison between two groups was done by the chi-squared test or Wilcoxon rank sum test. All tests were 2-sided, and a P value <0.05 was considered statistically significant.

## Results

### Study flow

**Figure 1:**
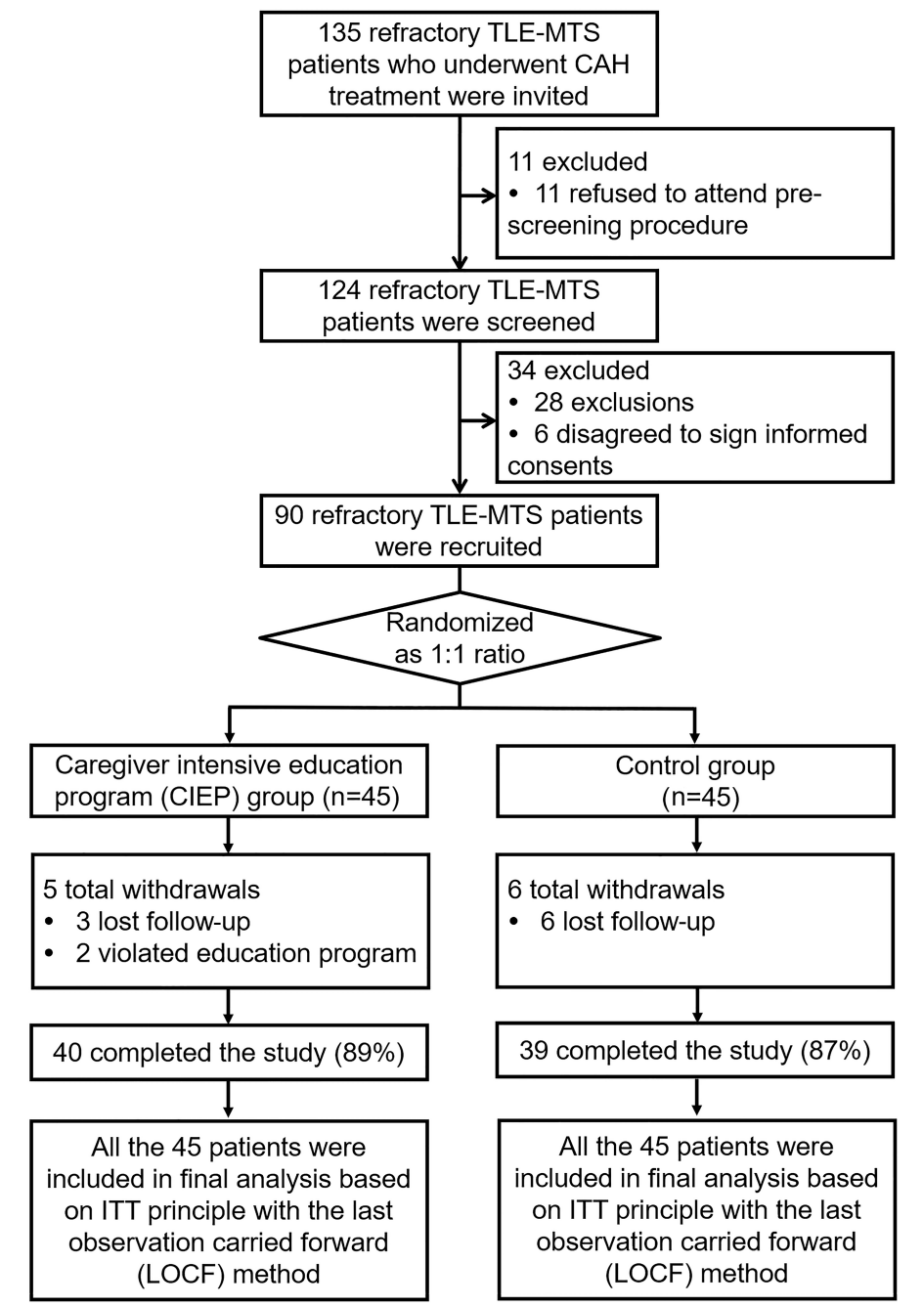
Study flow. TLE-MTS: temporal lobe epilepsy and mesial temporal sclerosis; CAH: cortico-amygdalohippocampectomy: ITT: intention-to-treat.

A hundred and thirty-five TLE-MTS patients who underwent CAH treatment were initially invited to our study, and 11 patients were excluded because they refused to attend the pre-screening procedure (Figure 1). After that, the remaining 124 patients were screened for eligibility, and 34 patients were excluded, which consisted of 28 patients who did not meet the inclusion criteria or met the exclusion criteria, and 6 patients who refused to sign the informed consents. Subsequently, 90 patients were recruited for the study, who were randomly allocated into two groups at a 1:1 ratio: CIEP group (n=45) and control group (n=45). In the CIEP group, there were 5 withdrawals (3 patients who were lost to follow-up and 2 patients who violated the education program), therefore 40 (89%) patients in this group completed the study. In the control group, there were 6 withdrawals (6 patients who were lost to follow-up), leaving 39 (87%) patients who completed the study in this group. In addition, the 45 patients in the CIEP group and 45 patients in the control group were all included in the final analysis based on the ITT principle with the LOCF method.

### Characteristics of patients and their caregivers

No difference in characteristics of patients was found between the two groups (Table 2). The mean age at surgery was 37.9±10.9 years and 40.3±12.3 years in the CIEP group and control group, respectively (P=0.330). There were 22 (48.9%) females and 23 (51.1%) males in the CIEP group, and 27 (60.0%) females and 18 (40.0%) males in the control group (P=0.290). For education level, the number of patients with the highest education being primary school or less, high school, undergraduate, and graduate or above were 10 (22.2%), 18 (40.0%), 15 (33.3%) and 2 (4.5%), respectively, in the CIEP group, and 11 (24.4%), 21 (46.7%), 8 (17.8%), and 5 (11.1%), respectively, in the control group (P=0.296). In addition, the mean disease duration was 27.4±9.2 years and 28.6±9.5 years (P=0.553), respectively, in the two groups. Other information of patients' characteristics is presented in Table 2.

With respect to the caregivers, no difference of caregivers' characteristics was found between the CIEP group and the control group. The mean age of caregivers was 45.0±11.6 years in the CIEP group and 43.8±10.0 years in the control group (P=0.588). The number of females and males was 25 (55.6%) and 20 (44.4%) in caregivers of the CIEP group, and 23 (51.1%) and 22 (48.9%) in caregivers of the control group (P=0.673). The number of caregivers with the highest education being primary school or less, high school, undergraduate, and graduate or above was 6 (13.3%), 18 (40.0%), 16 (35.6%), and 5 (11.1%), respectively, in the CIEP group, and 3 (6.6%), 22 (48.9%), 16 (35.6%), and 4 (8.9%), respectively, in the control group (P=0.680). The mean MMSE score of caregivers was 28.8±0.6 and 28.6±0.7, respectively, in the two groups (P=0.227).

### Baseline levels of anxiety, depression, and QOL

The HADS-A score (P=0.878), percentage of patients with anxiety (P=0.832), anxiety severity (P=0.302), HADS-D score (P=0.788), percentage of patients with depression (P=1.000), depression severity (P=0.781), and QOLIE-31 score (P=0.931) at baseline were similar between the two groups (Table 3). These data indicated that there was no difference of anxiety, depression or QOL between the groups at baseline.

**Table 1:** Themes in the educational sessions of caregiver intensive education program (CIEP).

Themes	Description of contents
1. Basic knowledge	
	(i) causes and pathophysiology of epilepsy and different types of seizures
	(ii) prevalence and incidence of epilepsy
	(iii) famous people with epilepsy and what they had achieved
	(iv) important diagnostic tests including electroencephalography and magnetic resonance imaging
	(v) pre-seizures symptoms, post-seizures developments, and occurrence
	(vi) importance of an accurate observation, documentation, and description of seizures
2. Therapy	
	(i) major aspects of therapy, such as current treatments, aims of therapy, the need for active cooperation
	(ii) therapy efficacy assessment
3. Drug management	
	(i) overview of antiepileptic drugs (AEDs)
	(ii) options of drugs
	(iii) guidelines for pharmacotherapy
	(iv) importance of long-term, regular medicine use
	(v) administration of adverse events by AEDs
4. Prognosis	
	(i) chances of entering remission
	(ii) chances and risks of discontinuation of AEDs after achieving seizures control
	(iii) possibilities and strategies in the case of continuing seizures
5. Prevention	
	(i) recognition and avoidance of individual seizures-provoking factors
	(ii) attention should be paid to the precursors before seizures
6. Seizures management	
	(i) better ways to cope with epilepsy
	(ii) safety treatment measures after epileptic seizures of patients
	(iii) necessary equipment to prevent accidents
7. Appropriate exercise	
	(i) influence of epilepsy on the personality of patients
	(ii) persist in exercise and matters needing attention in exercise
8. Psychological care	
	(i) influence of epilepsy on the personality of patients
	(ii) symptoms of stress
	(iii) coping with anxiety or depression
	(iv) building confidence for patients
9. Psychosocial support	
	(i) importance of family companionship, understanding, and care
	(ii) importance of recognition and acceptance form friends and social contacts
10. Lifestyle	
	(i) development a healthy and nutritious diet for patients
	(ii) strategies to help patients quit smoking and drinking
11. Social activity and life	
	(i) meaningful social activities contributing to keep patients optimistic and positive
	(ii) support for professional life
12. Relationships	
	(i) overview of relationships
	(ii) management of relationships after epilepsy occurrence

**Table 2:** Characteristics of patients with drug-resistant refractory temporal lobe epilepsy and mesial temporal sclerosis (TLE-MTS) and their caregivers.

Characteristics	CIEP group (n=45)	Control group (n=45)	P value
**Patients**			
Age at surgery (years), mean±SD	37.9±10.9	40.3±12.3	0.330
Gender, n (%)			0.290
Female	22 (48.9)	27 (60.0)	
Male	23 (51.1)	18 (40.0)	
Marital status, n (%)			0.512
Married	30 (66.7)	27 (60.0)	
Single	15 (33.3)	18 (40.0)	
Highest education, n (%)			0.296
Primary school or less	10 (22.2)	11 (24.4)	
High school	18 (40.0)	21 (46.7)	
Undergraduate	15 (33.3)	8 (17.8)	
Graduate or above	2 (4.5)	5 (11.1)	
Employment status, n (%)			0.803
Employed	34 (75.6)	35 (77.8)	
Unemployed	11 (24.4)	10 (22.2)	
Age at epilepsy onset (years), mean±SD	10.5±7.8	11.7±6.9	0.431
Disease duration (years), mean±SD	27.4±9.2	28.6±9.5	0.553
Family history of epilepsy, n (%)	10 (22.2)	10 (22.2)	1.000
Family history of psychiatric disorders, n (%)	6 (13.3)	5 (11.1)	0.748
Complications, n (%)			
Hypertension	5 (11.1)	6 (13.3)	0.748
Hyperlipidemia	4 (8.9)	5 (11.1)	0.725
Diabetes	2 (4.5)	2 (4.5)	1.000
Seizure frequencies (times per month), mean±SD	5.6±1.7	6.0±1.7	0.277
Presence of febrile seizures, n (%)	3 (6.6)	7 (15.6)	0.180
Presence of left-sided MTS, n (%)	28 (62.2)	30 (66.7)	0.660
Disorganized VEEG background activity, n (%)	9 (20.0)	13 (28.9)	0.327
Asymmetric VEEG background activity, n (%)	9 (20.0)	10 (22.2)	0.796
Contralateral slow-waves on VEEG, n (%)	10 (22.2)	12 (26.7)	0.624
Contralateral epileptiform discharges on VEEG, n (%)	10 (22.2)	13 (28.9)	0.468
**Caregivers**			
Age (years), mean±SD	45.0±11.6	43.8±10.0	0.588
Gender, n (%)			0.673
Female	25 (55.6)	23 (51.1)	
Male	20 (44.4)	22 (48.9)	
Highest education, n (%)			0.680
Primary school or less	6 (13.3)	3 (6.6)	
High school	18 (40.0)	22 (48.9)	
Undergraduate	16 (35.6)	16 (35.6)	
Graduate or above	5 (11.1)	4 (8.9)	
MMSE score, mean±SD	28.8±0.6	28.6±0.7	0.227

Student's *t*-test or chi-squared test were used for statistical analyses. CIEP: caregiver intensive education program; SD: standard deviation; VEEG: video electroencephalogram; MMSE: mini-mental state examination.

**Table 3:** Anxiety, depression, and quality of life at baseline (M0).

Items	CIEP group (n=45)	Control group (n=45)	P value
HADS-A score, mean±SD	7.0±3.1	7.1±3.7	0.878
Anxiety patients, N (%)	20 (44.4)	19 (42.2)	0.832
Anxiety severity, N (%)			0.302
Mild	16 (35.6)	12 (26.7)	
Moderate	2 (4.5)	5 (11.1)	
Severe	2 (4.5)	2 (4.5)	
HADS-D score, mean±SD	6.4±3.0	6.5±3.3	0.788
Depression patients, N (%)	12 (26.7)	12 (26.7)	1.000
Depression severity, N (%)			0.781
Mild	8 (17.8)	8 (17.8)	
Moderate	4 (8.9)	2 (4.5)	
Severe	0 (0.0)	2 (4.5)	
QOLIE-31 score, mean±SD	53.1±13.9	53.4±12.8	0.931

Student's *t*-test, chi-squared test, or Wilcoxon rank sum test were used for statistical analyses. CIEP: caregiver intensive education program; HADS-A: Hospital Anxiety and Depression Scale-anxiety; SD: standard deviation; HADS-D: Hospital Anxiety and Depression Scale-depression; QOLIE: quality of life in epilepsy.

### Effect of CIEP on the amelioration of anxiety

**Figure 2:**
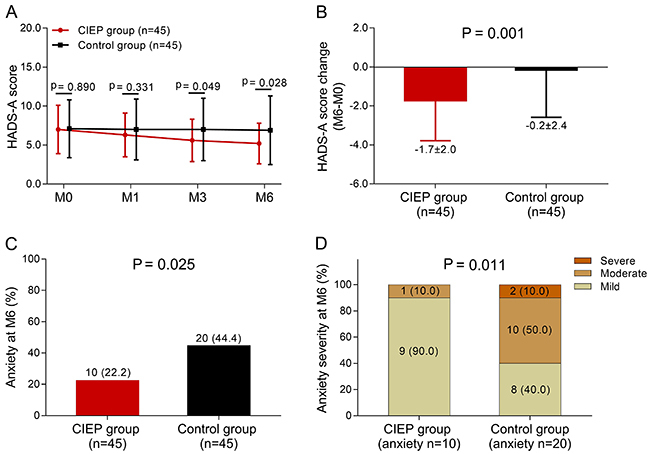
Effect of caregiver intensive education program (CIEP) on anxiety in patients with drug-resistant temporal lobe epilepsy and mesial temporal sclerosis (TLE-MTS) who underwent cortico-amygdalohippocampectomy (CAH). **A**, HADS-A score at month (M)0, M1, M3, and M6 in the CIEP group and control group; **B**, HADS-A score change (M6-M0) in the two groups. Data are reported as means±SD. **C**, Percentage of patients with anxiety in the two groups and **D**, percentage of anxiety severity in the two groups. P<0.05 was considered statistically significant by Student's *t*-test, Wilcoxon rank sum test, or chi-squared test. HADS-A: Hospital Anxiety and Depression Score-anxiety.

The HADS-A score decreased in the CIEP group compared to the control group at M3 (P=0.049) and M6 (P=0.028); there was no difference at M1 (P=0.331) (Figure 2A). The HADS-A score change (M6-M0) (P=0.001) (Figure 2B) and proportion of patients with anxiety at M6 (P=0.025) (Figure 2C) were decreased in the CIEP group compared with the control group. Additionally, anxiety at M6 was less severe in the CIEP group compared to the control group (P=0.011) (Figure 2D), which indicated that CIEP reduced anxiety in patients with drug-resistant TLE-MTS who underwent CAH.

### Effect of CIEP on the amelioration of depression

**Figure 3:**
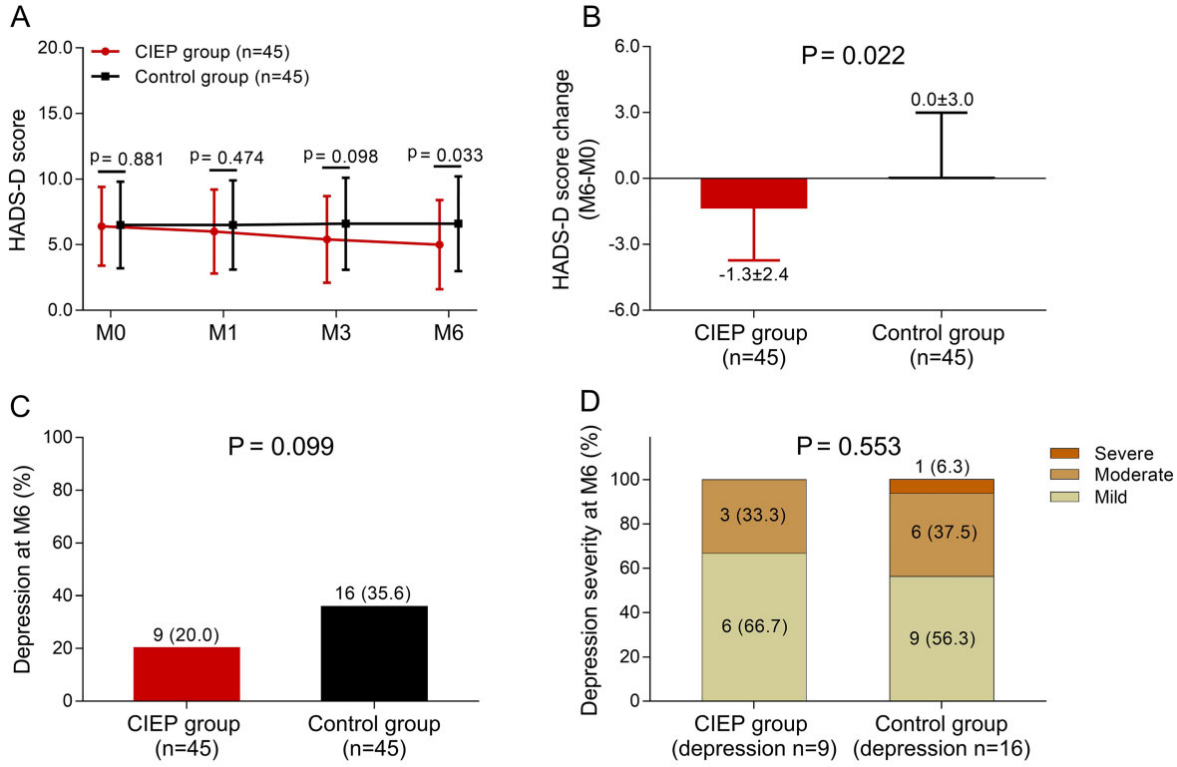
Effect of a caregiver intensive education program (CIEP) on depression in patients with drug-resistant temporal lobe epilepsy and mesial temporal sclerosis (TLE-MTS) who underwent cortico-amygdalohippocampectomy (CAH). **A**, HADS-D score at month (M)0, M1, M3, and M6 in the CIEP group and control group; **B**, HADS-D score change (M6-M0) in the two groups. Data are reported as means±SD. **C**, Percentage of patients with depression in the two groups and **D**, percentage of depression severity in the two groups. P<0.05 was considered statistically significant by the Student's *t*-test, Wilcoxon rank sum test, or chi-squared test. HADS-D: Hospital Anxiety and Depression Score-depression.

The HADS-D score was decreased at M6 (P=0.033), but showed no difference between groups at M1 (P=0.474) or M3 (P=0.098) (Figure 3A). The HADS-D score change (M6-M0) was reduced compared to the control group (P=0.022) (Figure 3B). However, no difference of proportion of patients with depression (P=0.099) (Figure 3C) or depression severity (P=0.553) (Figure 3D) at M6 was found between the two groups. These results indicated that CIEP ameliorated depression in patients with drug-resistant TLE-MTS who underwent CAH.

### Effect of CIEP on QOL improvement

**Figure 4:**
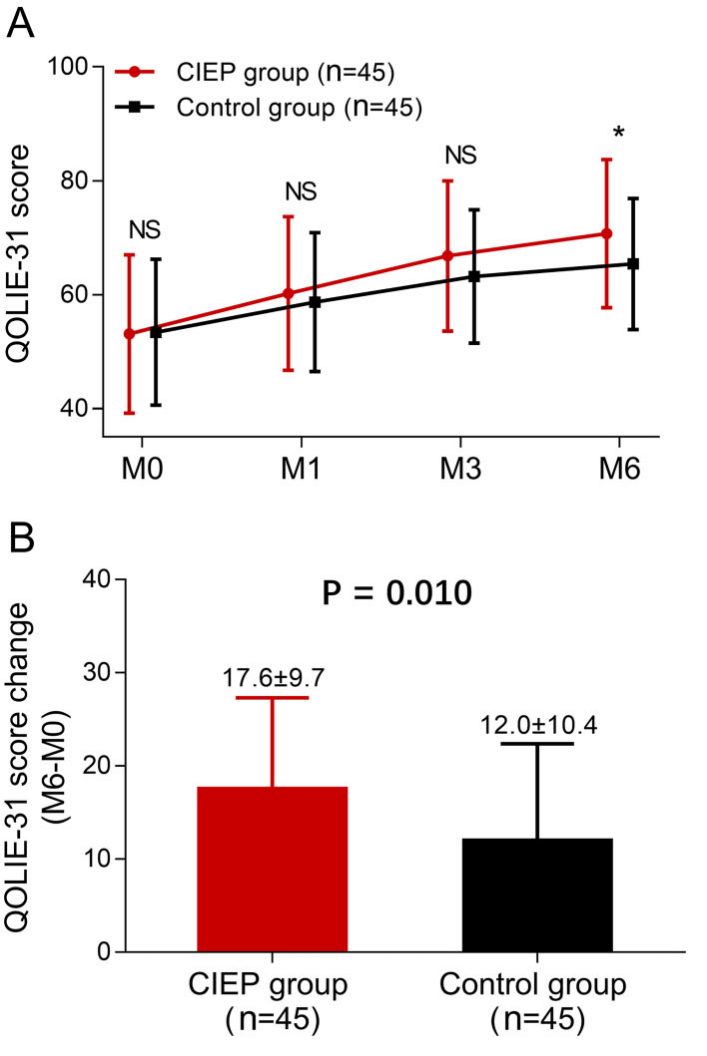
Effect of the caregiver intensive education program (CIEP) on the quality of life (QOL) of patients with drug-resistant temporal lobe epilepsy and mesial temporal sclerosis (TLE-MTS) who underwent cortico-amygdalohippocampectomy (CAH). **A**, QOLIE-31 score at month (M)0, M1, M3, and M6 in the CIEP group and control group; **B**, QOLIE-31 score change (M6-M0) in the two groups. Data are reported as means±SD. P<0.05 was considered statistically significant by Student's *t*-test. NS: not significant; QOLIE-31: Quality of Life in Epilepsy Inventory-31.

The QOLIE-31 score was higher in the CIEP group than in the control group at M6 (P=0.043), while there was no difference between the two groups at M1 (P=0.582) or M3 (P=0.174) (Figure 4A). QOLIE-31 score change (M6-M0) was also increased in the CIEP group compared with the control group (P=0.010) (Figure 4B). These data suggested that CIEP improved the QOL in patients with drug-resistant TLE-MTS who underwent CAH.

### Effect of CIEP on Engel class

**Figure 5:**
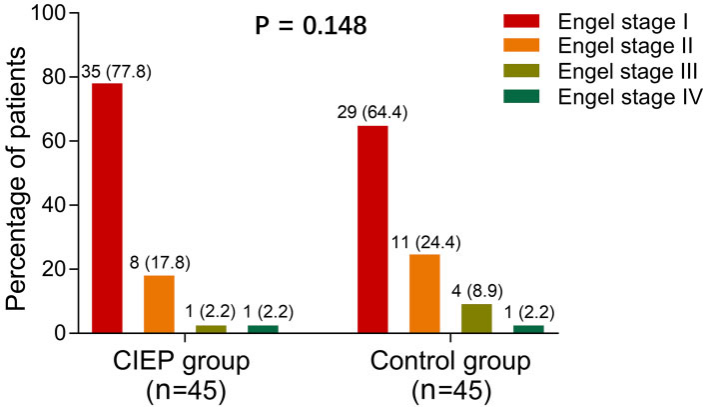
Engel classes and percentages of the caregiver intensive education program (CIEP) group and the control group at month 6. P<0.05 was considered statistically significant by the Wilcoxon rank sum test.

Treatment efficacy was compared between groups at M6. The CIEP group showed numerically lower Engel classes compared with the control group but without statistical significance (P=0.148) (Figure 5). This result suggested that the CIEP seemed to have less influence on improving treatment efficacy in patients with drug-resistant TLE-MTS who underwent CAH.

## Discussion

The CIEP program was an educational program specifically designed for the caregivers of patients with drug-resistant TLE-MTS, which started with a brief introduction of the program to both caregivers and patients and followed by a series of professional education of the disease, guidance of disease management, and help with problem solving and appointment scheduling among caregivers. In this study, we discovered that i) CIEP ameliorated anxiety and depression in patients; ii) CIEP improved QOL in patients; and iii) CIEP had less effect on improving treatment efficacy in patients.

Anxiety and depression are common complications in patients with epilepsy, especially in patients with TLE-MTS owing to the specific lesion area in the brain, and these psychiatric disorders have been reported to play harmful roles in patient prognosis (14,15). However, very little investigation has been done to study the effect of a psychological intervention on improving anxiety and depression in patients with drug-resistant TLE-MTS; most of the studies are conducted on other types of patients with epilepsy. For instance, a previous randomized study elucidates that systematic family therapy (group psychotherapy treatment with family as a unit) combined with antiepileptic drugs is more effective than antiepileptic drugs alone in treating epilepsy and alleviating anxiety and depression in adolescents (16). Another single-group trial reports that an internet-delivered and transdiagnostic program mitigates anxiety, depression, epilepsy-specific depression, and disability in adults with epilepsy (17). These previous studies indicate that professional intervention programs are effective in reducing anxiety and depression in epilepsy. As for patients with drug-resistant TLE-MTS, a previous non-randomized controlled study elucidates that a cognitive-behavioral therapy-based group psychological intervention improves QOL and reduces the depression and anxiety symptoms in such patients (18). Although this previous study and our study were both conducted in patients with drug-resistant TLE-MTS, this previous study is still distinctive compared to our study in the following aspects: a) only a part of the patients in their study received CAH, while all the patients in our study were treated by CAH; b) the intervention program in their study was performed on patients but not caregivers; and c) the content of their intervention program was different from ours. In this study, we conducted a program named CIEP for caregivers aiming at ameliorating anxiety and depression as well as improving QOL in patients and found that anxiety and depression were both ameliorated more effectively by CIEP compared with conventional care (control). Several explanations are possible: i) the education for caregivers in CIEP was more intensive, professional, and comprehensive compared with that in conventional care, which indicated that the caregivers receiving CIEP might have better efficacy in learning and practicing the knowledge about patients care and managing mood disorders; ii) the education for caregivers in CIEP contained a broader content compared with that in conventional care, including giving instructions and explanations of refractory TLE-MTS, problems consulting, recording of medicine use, epilepsy seizure status tracking, and doctor appointment scheduling; and iii) the CIEP program focused more on resolving the problems that caregivers met in the daily care of patients, giving instructions to the caregivers on medications that patients take and helping with making appointments for outpatient service, which was more targeted and effective compared with conventional care.

The majority of the patients in our study were facing a decrease in QOL, which was partially due to the high prevalence of psychiatric disorders including anxiety and depression post-CAH (19,20). Strategies for improving QOL in patients with drug-resistant TLE-MTS post-CAH have not been reported yet, and most studies are conducted in different patient cohorts. For instance, a previous clinical randomized trial demonstrates that a cognitive behavioral intervention program (Home-Based Self-management and Cognitive Training Changes Lives - HOBSCOTCH program) markedly improves the QOL in adult patients with epilepsy (21). Another study reports that a community-based epilepsy awareness program improves QOL in patients with epilepsy presenting as decreased depression, fewer memory difficulties, less work/social issues, and reduced seizure worry (22). To the best of our knowledge, this study was the first to investigate the effect of a specific educational program designed for caregivers aiming at improving the QOL of patients with drug-resistant TLE-MTS who underwent CAH. In this study, we found that CIEP markedly improved the QOL of patients, which might be due to: i) the education regarding appropriate exercise, lifestyle, social activity, and life and relationships in caregivers aiming at improving the QOL of patients; ii) the content of CIEP aimed to help solving problems that caregivers met in the daily care of patients, including the problems of QOL, which might also contribute to the QOL improvement in patients; and iii) more severe depression/anxiety was associated with worse QOL, and the patients in the CIEP group showed a decrease in depression as well as anxiety, which subsequently improved the QOL due to a healthier psychiatric status of patients (23).

There were several limitations in this study: i) the study was not blinded, thus there might be observer bias; however, our study aimed to evaluate the effect of an educational program, which was difficult to carry out with a blinding method; ii) the follow-up duration in our study was relatively short, which should be prolonged in future studies; and iii) the psychiatric status of caregivers in our study was not assessed, which might have an influence on patient mood disorders and QOL.

In conclusion, the CIEP ameliorated anxiety and depression as well as improved QOL in patients with drug-resistant TLE-MTS who underwent CAH. This provided evidence for the potential application of caregiver programs in improving patient psychiatric outcomes and QOL in clinical practice.

## References

[1] Fisher RS, van Emde Boas W, Blume W, Elger C, Genton P, Lee P (2005). Epileptic seizures and epilepsy: definitions proposed by the
International League Against Epilepsy (ILAE) and the International Bureau
for Epilepsy (IBE). Epilepsia.

[2] Morimoto K, Fahnestock M, Racine RJ (2004). Kindling and status epilepticus models of epilepsy: rewiring the
brain. Prog Neurobiol.

[3] Engel J, Wiebe S, French J, Sperling M, Williamson P, Spencer D (2003). Practice parameter: temporal lobe and localized neocortical
resections for epilepsy. Epilepsia.

[B04] Filho GM, Mazetto L, Gomes FL, Marinho MM, Tavares IM, Caboclo LO (2012). Pre-surgical predictors for psychiatric disorders following
epilepsy surgery in patients with refractory temporal lobe epilepsy and
mesial temporal sclerosis. Epilepsy Res.

[5] Yang W, Chen C, Wu B, Yang Q, Tong D (2019). Comprehensive analysis of presurgical factors predicting
psychiatric disorders in patients with refractory temporal lobe epilepsy and
mesial temporal sclerosis underwent
cortico-amygdalohippocampectomy. J Clin Lab Anal.

[6] Puka K, Widjaja E, Smith ML (2017). The influence of patient, caregiver, and family factors on
symptoms of anxiety and depression in children and adolescents with
intractable epilepsy. Epilepsy Behav.

[7] Fong CY, Hong SY, Ong LC, Lim WK, Lua PL (2019). Improving awareness, knowledge, and attitude among Malaysian
parents of children with epilepsy using an Interactive Animated Epilepsy
Education Programme (IAEEP). Epilepsy Behav.

[8] Meyer B, Weiss M, Holtkamp M, Arnold S, Bruckner K, Schroder J (2019). Effects of an epilepsy-specific Internet intervention (Emyna) on
depression: Results of the ENCODE randomized controlled
trial. Epilepsia.

[9] Proposal for revised classification of epilepsies, epileptic syndromes (1989). Commission on Classification and Terminology of the International
League Against Epilepsy. Epilepsia.

[10] Labate A, Ventura P, Gambardella A, Le Piane E, Colosimo E, Leggio U (2006). MRI evidence of mesial temporal sclerosis in sporadic “benign”
temporal lobe epilepsy. Neurology.

[11] Zigmond AS, Snaith RP (1983). The hospital anxiety and depression scale. Acta Psychiatr Scand.

[12] Cramer JA, Perrine K, Devinsky O, Bryant-Comstock L, Meador K, Hermann B (1998). Development and cross-cultural translations of a 31-item quality
of life in epilepsy inventory. Epilepsia.

[13] Engel J, VanNess P, Rasmussen T, Ojemann L (1993). Outcome with respect to epileptic seizures surgical treatment of the
epilepsies.

[14] Shcherbakova N, Rascati K, Brown C, Lawson K, Novak S, Richards KM (2014). Factors associated with seizure recurrence in epilepsy patients
treated with antiepileptic monotherapy: a retrospective observational cohort
study using US administrative insurance claims. CNS Drugs.

[15] de Oliveira GN, Kummer A, Salgado JV, Portela EJ, Sousa-Pereira SR, David AS (2010). Psychiatric disorders in temporal lobe epilepsy: an overview from
a tertiary service in Brazil. Seizure.

[16] Li J, Wang X, Meng H, Zeng K, Quan F, Liu F (2016). Systemic family therapy of comorbidity of anxiety and depression
with epilepsy in adolescents. Psychiatry Investig.

[17] Gandy M, Karin E, Fogliati VJ, McDonald S, Titov N, Dear BF (2016). A feasibility trial of an Internet-delivered and transdiagnostic
cognitive behavioral therapy treatment program for anxiety, depression, and
disability among adults with epilepsy. Epilepsia.

[18] de Barros ACS, Furlan AER, Marques LHN, de Araujo GM (2018). Effects of a psychotherapeutic group intervention in patients
with refractory mesial temporal lobe epilepsy and comorbid psychogenic
nonepileptic seizures: a nonrandomized controlled study. Seizure.

[19] Filho GM, Mazetto L, da Silva JM, Caboclo LO, Yacubian EM (2011). Psychiatric comorbidity in patients with two prototypes of focal
versus generalized epilepsy syndromes. Seizure.

[20] Tellez-Zenteno JF, Wiebe S (2008). Prevalence of psychiatric disorders in patients with epilepsy: what we
think we know and what we know.

[21] Caller TA, Ferguson RJ, Roth RM, Secore KL, Alexandre FP, Zhao W (2016). A cognitive behavioral intervention (HOBSCOTCH) improves quality
of life and attention in epilepsy. Epilepsy Behav.

[22] Giuliano L, Cicero CE, Padilla S, Rojo Mayaregua D, Camargo Villarreal WM, Sofia V (2019). Knowledge, stigma, and quality of life in epilepsy: Results
before and after a community-based epilepsy awareness program in rural
Bolivia. Epilepsy Behav.

[23] Izci F, Findikli E, Camkurt MA, Tuncel D, Sahin M (2016). Impact of aggression, depression, and anxiety levels on quality
of life in epilepsy patients. Neuropsychiatr Dis Treat.

